# Cultural Adaptation of Community Informant Tool for Detection of Maternal Depression in Rural Pakistan

**DOI:** 10.3389/fpsyt.2021.598857

**Published:** 2021-04-01

**Authors:** Shamaila Mohsin, Najia Atif, Waqas Rabbani, Ahmaren Tariq, Shahzad Ali Khan, Mahjabeen Tariq, Siham Sikander

**Affiliations:** ^1^Department of Community Medicine, Army Medical College, National University of Medical Sciences, Rawalpindi, Pakistan; ^2^Human Development Research Foundation, Islamabad, Pakistan; ^3^Department of Behavioural Sciences, Shifa College of Medicine, Islamabad, Pakistan; ^4^Global Health Department, Health Services Academy, Islamabad, Pakistan; ^5^University of Liverpool, Liverpool, United Kingdom; ^6^Primary Care and Mental Health, University of Liverpool, Liverpool, United Kingdom

**Keywords:** cultural adaptation, community informant, detection tool, maternal depression, Pakistan, Proactive Case Detection

## Abstract

**Background:** Evidence indicates that mental health issues like depression, epilepsy, and substance misuse can be detected with reasonable accuracy in resource-poor settings. The Community Informant Detection Tool (CIDT) is one such approach used for detecting mental health problems, including depression. We adapted this community informant approach for detecting maternal depression in Pakistan.

**Methods:** Adaptation of Community Informant Detection Tool for Maternal Depression (CIDT-MD) involved five steps. First, a scoping review of the literature was conducted to select an appropriate tool for adaptation. Second, in-depth interviews were conducted to explore the idioms of depression and distress, perceived causes, and the effects of maternal depression among currently depressed and recovered mothers (*n* = 11), mothers in law (*n* = 6), and Primary Care Providers (Primary Care Physicians and Lady Health Supervisors) (*n* = 6). Third, case vignettes and illustrations were created with input from a panel of mental health experts, incorporating the idioms of depression and distress used, causes, and effects for each symptom described. Fourth, to assess the comprehensibility of the illustrations and level of understanding, Focus Group Discussions (*n* = 4) were done with purposely selected community health workers (Lady Health Workers and Lay Peers, *n* = 28) trained in delivering maternal depression intervention. The final step was reflection and inputs by a panel of mental health experts on all steps to finalize the content of the tool.

**Results:** Context-specific cultural adaptation in the presentation and format of CIDT-MD was conducted successfully. Lady Health Workers (LHW) and Lay Peers (LP) were found to be the most appropriate persons to use the tool and function as the informants. The adapted tool with all its vignettes and illustrations was found to be easily understandable, comprehensible, and culturally appropriate, meaningful, and contextually relevant by the community health workers and peers working in the relevant settings. They easily relate to and identify potentially depressed such women lining up with the tool. Lastly, the coding of the tool was found easy to follow as well.

**Conclusion:** The Community Informant Detection Tool for Maternal Depression (CIDT-MD) is a culturally acceptable, easy to use, and comprehensible tool for detecting maternal depression in community settings of Pakistan. The community informants found the content and approach highly relevant to the local needs.

## Introduction

The burden of maternal depression is highest in the South Asian region as indicated by a recent systematic review (1). Early detection of maternal depression is a public health priority to address its direct effects on the woman and her child as well as arrest its inter-generational impact (2). However, maternal depression largely remains undetected and under-recognized with symptoms persisting well-beyond the perinatal period (3). Moreover, it is a major contributor to the burden of suicide, which is among the leading causes of death in young women of reproductive age (4).

World Mental Health Atlas indicates that there exists a large treatment gap (the difference between the number of women who need care and those who receive care) for mental health issues in Low Middle-Income Countries (LMIC). The median number of qualified mental health personnel in LMIC per 100,000 population is only 6.2 (5). Research indicates that with limited specialists within LMIC, there has been a rise of task shifting through non-specialists, such as primary health-care staff (PHC) and lay community health workers (CHW), to address the huge treatment gap (6, 7). It includes both the detection and management of mental health issues, especially the Common Mental Disorders (CMD) (8). Task-shifting has its evidence-based advantages of greater coverage, being cost-effective, local non-specialists being more knowledgeable about local idioms of expression and experiences of psychological distress, and can markedly reduce stigma (9–12).

Timely detection of CMDs is a challenge. Integration of mental health into primary care and its use of screening tools have been advocated for early detection (13). Despite this evidence, many women in LMIC settings have accessibility issues to the primary care set up (14, 15). Thus, innovation is needed to help early detection.

One such innovative approach is to use the community-based informants for proactive case detection. Using this approach the Community Informant Detection Tool (CIDT) for mental disorders was developed in Nepal (16). The community informants used the tool in their routine activities. The CIDT had paragraph-long vignettes (aided with pictures) accompanied with a simple 5-point Likert scale to be matched to a “probable positive case” in the community. In case the person in the community had significant features of the description, then the informant answered two additional questions in the tool: whether the identified person was perceived to have impaired daily functioning and if the person required support. In case of significant matching and a positive response to at least one of the additional questions, the “probable positive case” identified through the tool was then encouraged to visit the local health facility (16).

However, the CIDT was developed for women living in Nepal and the women's lives reflected in the pictorial illustrations depict adherence to socio-cultural norms, including appearance, clothing, participation in rural domestic work, and income-generating activities outside the household. Furthermore, it was used for detecting depression, epilepsy, substance use disorders, and conduct disorders. Each disorder had a description in local lay terms. The tool developed in another setting may not capture the cultural context where the meaning and experience of symptoms may differ. Furthermore, there was a need to adapt the tool for detecting maternal depression in similar settings. Therefore, for the cultural adaptation and appropriateness, a five-step qualitative approach was taken.

## Materials and Methods

### Study Setting

The study was conducted in one of the seven rural sub-districts of the District Rawalpindi, called Kallar Syedan in Punjab, Pakistan. It has 11 Union Councils (UCs). Each UC is the smallest administrative unit consisting of 15–20 villages and has a population of about 22,000–25,000. Each UC has a primary health care facility called the Basic Health Unit (BHU). There is an adequate number (*n* = 122) of Lady Health Workers (LHWs) in the area. The average household consists of 6.2 members. The spoken and written language in the study area is Urdu. The sub-district is representative of a typical low-socioeconomic rural area of Pakistan. This area was selected for several methodological and logistic reasons as it is geographically, culturally, and socio-economically homogenous with similarities to other districts of Punjab. It is one of the first Pakistani districts where WHO mhGAP has been rolled out.

### Ethical Approval

The Ethical Review Board of Health Services Academy reviewed and approved the study (HSA) (No.7-82/IERC-HSA/2018-014). Information sheets explaining the objective, procedure, benefit, and confidentiality of the data and informed consent were given to all participants 24 h before data collection. For the illiterate, verbal and thumbprint consent was acquired in the presence of an eyewitness, and a family member also signed in their attendance.

### Study Procedure

A five-step process was conducted to adapt the CIDT-MD and evaluate its understanding and willingness to use perceived advantages and barriers.

#### Step 1: Selection of the Instrument

The focus in the first step was to select an appropriate instrument for proactive case detection of maternal depression. Experts from the disciplines of public and mental health conducted a scoping review of literature based on which a tool was selected (Mohsin et al., under review). Based on the cultural appropriateness, soundness in methodology, and the evidence of its accuracy in a particular cultural setting, the CIDT was selected. After the selection process, a collaborative iterative qualitative adaptation methodology was mapped out with the experts and the principal researchers.

#### Step 2: In-Depth Interviews With Key Informants

First, In-Depth Interviews (IDI) were conducted with a varied set of participants, purposively selected, ensuring all relevant stakeholders [mothers, mothers-in-law, and Primary Care Providers (Primary Care Physicians and Lady Health Supervisors)] were represented. These participants were approached to explore the local idioms of expression used to describe symptoms of maternal depression (including suicide), the chronicity of maternal depression, and associated issues such as domestic violence, poverty, and any other perceived causes and potential effects of maternal depression in their own language Urdu. Depressed mothers (Patient Health Questionnaire [PHQ] Score >10) and mothers who have recovered with a child <3 years old were identified. Mothers experiencing psychosis or suicidal ideation or any serious medical conditions were excluded. A topic guide was developed to address the research questions. It had open-ended questions about the idioms, perceived causes and effects of symptoms on the participants based on the *DSM-5* criteria for major depression. It included (i) difficulty in sleeping, (ii) loss of appetite, (iii), agitation, (iv) lack of concentration, (v) helplessness, (vi) fatigue, (vii) loss of interest, (viii) low mood and (ix) suicidal ideation.

Public health professionals and trained psychologists working in the study settings (SM, MT, and AT) who were bilingual and fluent in the local dialect (Urdu) collected the data. The team received training on conducting qualitative interviews. All the interviews were audio-recorded, while the duration ranged from 25–35 min. All the interviews were conducted in the house for women and mothers-in-law or the primary health-care facility (called Basic Health Units), depending on the preference of the participants. The three local data-collectors conducted, transcribed, and translated the IDIs into English. SM and AT conducted and transcribed all the English interviews. Members of the data collection team also corroborated cross checks of the audio-files and transcripts.

There were 22 IDIs conducted as shown in Table 1. All the mothers (100%) in the sample were married. The average age of the mother was 31.6 years (SD = 3). The average number of children among the participants was three. The characteristics of the mothers are detailed in Table 1.

**Table 1:** Socio-demographic characteristics of the participants.

**Participant Reference No**	**Age**	**No of Children**	**Marital Status**	**Years of Schooling**	**Family Structure**
DM 01	30	4	M	12	Joint
DM 02	32	2	M	12	Joint
DM 03	36	4	M	8	Nuclear
DM 04	30	3	M	14	Nuclear
DM 05	33	4	M	10	Joint
DM 06	32	4	M	10	Joint
RM 01	30	4	M	12	Joint
RM 02	28	2	M	12	Joint
RM 03	36	4	M	8	Nuclear
RM 04	30	3	M	14	Nuclear
RM 05	31	4	M	10	Joint
MIL 01	49	4	M	5	Joint
MIL 02	55	5	M	8	Joint
MIL 03	56	4	M	8	Nuclear
MIL 04	60	6	M	-	Joint
MIL 05	52	5	M	5	Joint
MIL 06	60	4	M	8	Joint
LHS 01	42	3	M	12	Nuclear
LHS 02	38	4	M	12	Joint
LHS 03	47	3	M	10	Nuclear
PCP 04	25	-	M	14	Nuclear
PCP 05	26	1	M	14	Joint
PCP 06	47	3	M	14	Nuclear

*DM, depresses mother; RM, recovered mother; MIL, mother-in-law; LHS, lady health supervisor; PCP, primary care physician*.

#### Step 3: Expert Panel Review—Development of Draft Tool

The meeting with the expert panel was routinely conducted for assessing the constructs from the IDIs. First, the idioms of depression and associated distress were listed and clustered according to the frequency of usage. The idioms explaining depression and its symptoms were then ranked based on the frequency of usage by the IDI participants. Separate columns were made for the frequency reported by mothers (depressed and recovered) and health-care providers. The selection of idioms was included in the case-specific vignette selected by the expert panel.

Second, a case vignette integrating the prioritized terms/idioms, the perceived cause, and effects of maternal depression for each of the nine symptoms was written. Thoughts, feelings, and perceptions related to the symptoms were also included. The frequently reported perceived causes were also included to create context-specific case vignettes that included personnel narratives and field notes.

Third, a professional artist was hired to draw a pencil sketch for each symptom. The artist was an experienced individual who had drawn similar sketches for training manuals of maternal mental health interventions delivered in similar settings. The artist made the final pencil sketches in line with the case vignettes and the narrative description.

#### Step 4: Focus Group Discussions With End Users

To assess the comprehensibility, understanding of the symptoms, and pictorial illustrations among the potential end-users, the community informants (i.e., health-care workers and peers trained in delivering maternal depression intervention), FGDs were conducted with an average number of 5–6 participants. The key areas explored included (1) if the pictorial illustration had depicted all the necessary information of that symptom (any changes that were required), (2) willingness and ability to detect such women in their vicinity (community settings) based on these illustrations, (3) recall of any case in their vicinity who experienced similar symptoms, and (4) facilitators and barriers of using such illustrations to detect maternal depression.

**Figure 1:**
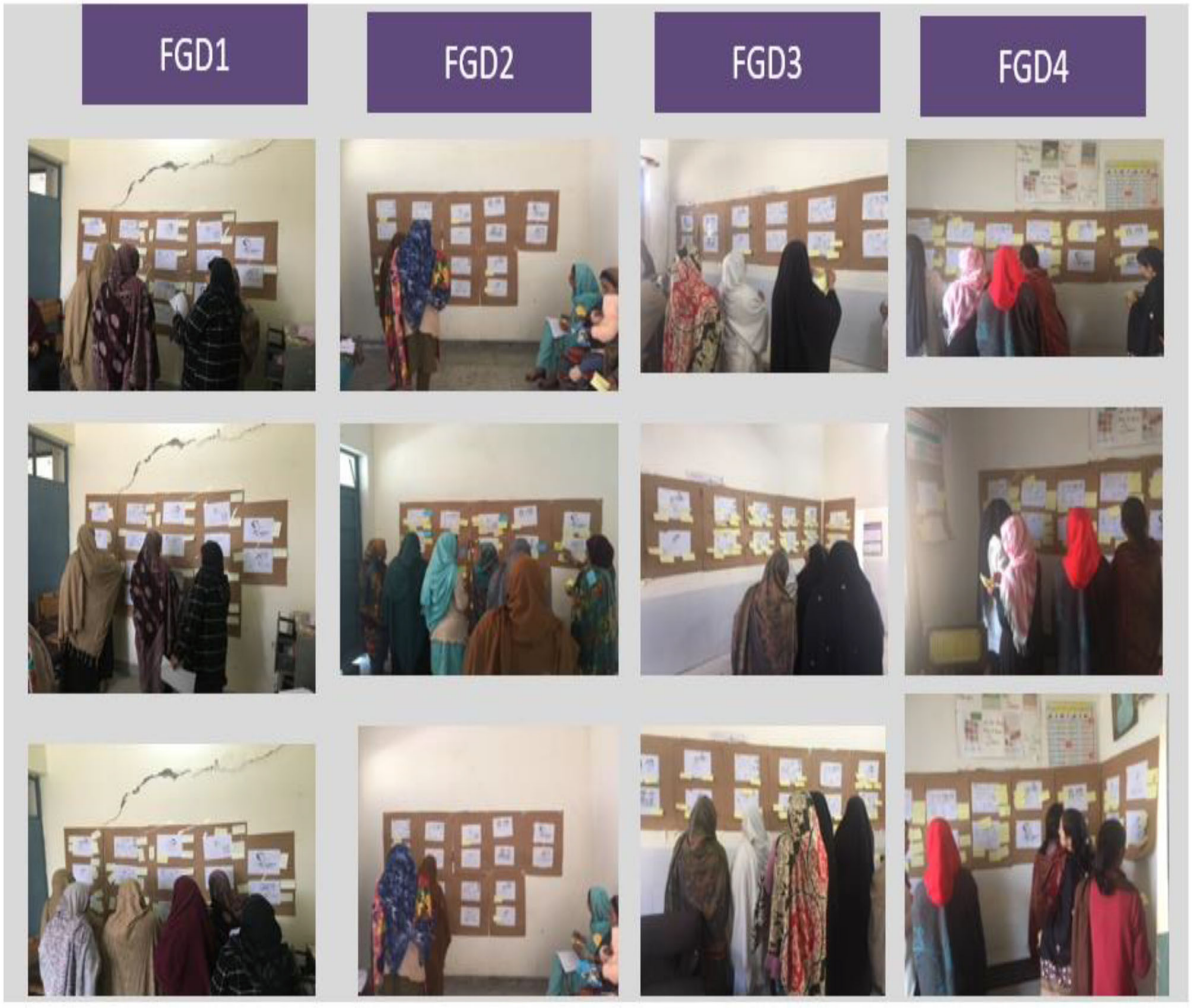
Card to pictorial illustration matching activity.

In the first half the participants were given two illustrated cards with idioms of depression and for each symptom were asked to match and paste the relevant cards on the pictorial illustrations, which were pasted on the wall as shown in Figure 1.

The second half of the FGDs facilitated the collection of narrative data to assess the level of understanding of the pictorial illustration by the participants. For each pictorial illustration, the lead author pointed it out and asked them if any changes were required, their acceptability and willingness to use, the recall of any mother with similar symptoms, advantages, and barriers of using such illustrations to detect maternal depression. This process was repeated for each illustration.

The FGDs were audio-taped, transcribed into the local language, and translated into English with quality checks from research assistants at the study site by a professional translator. Data collection and analysis were conducted simultaneously. The qualitative data were analyzed using a framework analysis approach (Ritchie and Spencer, 1994).

With an aim to finalize the prepared draft of CIDT-MD, FGDs (*n* = 4) were conducted with the community health service providers. They included both the LHWs (*n* = 15) and Peers (*n* = 13). The socio-demographic characteristics of the participants are listed in Table 2.

**Table 2:** Cause of maternal depression.

**S.No**	**Category**	**Sub-Theme**	**Quote (Source—interviews unless stated)**
1.	Interpersonal Conflict	1.1 Joint Family System	*I have been married for 8 years when I was 18 years of age I got married I was raised with a lot of love in my house I used to think I was the most adored daughter but when I came to my susral (in-laws) there everything was against my will I was ill-treated and now I have become a patient (IDI-DM06-3-16-20)*
		1.2 Unsupportive In-Laws	*The attitude of the in laws is not right with the mother mainly her mother in law and sister in law's she keeps on thinking what will they do next? And does not seem to concentrate. (IDI-DM01-1-24-25)*
		1.3 Uncaring Husband	*If there are quarrels with the husband every now and then over money matters then she cannot go to sleep at night and keeps thinking of the things he said to her and becomes sad and worried. (IDI-RM05-5-8-11)*
2.	Overwhelmed with domestic responsibility	2.1 Well Being of Children	*First there is one child then there is another then third and fourth then they have to get up early in the morning to go to the masjid to recite the suprarh (the Holy Quran) then they are school going then they are fed and bathed …there are hundreds of errands in a day that is why there is lack of concentration (IDI-RM02-2-5-7)*
		2.2 Multiple Caregiver Demands	*I am over burdened with the domestic chores there is a lot of work …one has to clean the whole house there is a large verandah then one has to cook the food then keep on cleaning the utensils all day long now I have a lot of issues in the house. (IDI-RM01-6-12-15)*
		2.3 Tend to Domestic Animals	*I was raised in Karachi and come here after marriage … in our house there are multiple errands we have kept goats in the house I feel so tired and exhausted looking after the house and the animals as mein bakri nal bakri bun gaye aan. (if I have become a goat myself with these goats). (IDI-RM06-6-12-15)*
3.	Physical ailments	3.1 Women Experiencing Ill Health	*I had TB I remained in bed for three months I would cough all day long I did not feel like eating anything I was very weak I could not take care of myself or my children. (IDI-DM06-2-14-16)*
		3.2 Ill Health of Family Members	*My husband had a heart attack it's been five months since he had two stents in the heart the day before yesterday he again developed chest pain now he is to have a heart by-pass operation I can't go to sleep at night thinking of it. (IDI-DM04-1-10-13)*
		3.3 Ailment of Children	*I think that the mother cannot go to sleep when the child is sick and cannot go to sleep if he has fever or pain in abdomen he will cry all night I think for me this the reason for difficulty in sleeping when my child is sick (IDI-RM04-1-10-13)*
4.	Poverty	4.1 Unmet Financial Needs	*If the husband is unemployed the expenses of the house are not met just as …my husband does not get the official job he is a laborer some days he finds work some days he does not then the I have to work extra hard in the house. (IDI-DM05-3-1-3)*
		4.3 Family Debt	*There are some who are in debt, if the debt is too much and one has been thinking too much, if there are no means of settling the debt, then the mother is very worried (IDI-LHS 03-6-10-13)*

#### Step 5: Final Draft Preparation Through Expert Panel Discussion

After incorporating findings from the FGDs, the draft of the CIDT-MD was handed over to the expert panel, which had experienced psychiatrists for review. The panel of experts comprised four experts from the field of child development, mental and public health. Two of the experts work and live in Pakistan while the other two lived abroad (in a Western country). They had a vast experience of research in the areas of perinatal, infant, child, and adolescent mental health, in low- and middle-income settings as Pakistan. One such researcher has pioneered task-shifting approaches for mental health (non-specialists delivering simplified but effective psychotherapy under the supervision of specialists). They were frequently consulted and briefed during the four-step procedure.

### Data Analysis

All the IDI were audio-taped, transcribed into the local language (Urdu), and translated into English, with quality checks from research assistants at the study site by a professional translator. Analysis of the data was conducted deductively with a framework analysis (FA) approach using MS Word and the NVIVO software. Framework analysis has five key steps: (1) coding (indexing), (2) developing a working analytical framework, (3) applying the analytical framework, (4) charting data into the framework matrix, and (5) mapping and interpreting the data. Using MS Word, the researcher read a set of transcripts from pilot interviews to identify the emerging themes and to initiate the development of a thematic framework. The research team evaluated the analysis to ensure that the interpretations were credible and valid.

## Results

### Step 2

#### Idioms of Depression and Its Symptoms

The idioms of depression for all the nine symptoms—(i) difficulty in sleeping, (ii) loss of appetite, (iii) agitation, (iv) lack of concentration, (v) helplessness, (vi) fatigue, (vii) loss of interest, (viii) low mood and (ix) suicidal ideation—were elicited and were incorporated into the vignettes.

#### Perceived Cause and Effect of Maternal Depression

The study participants attributed depression to four main themes in their community. The most frequent of these were Inter-personnel Conflicts, Domestic Abuse, Poverty and Dependence, and Physical Ailments. It was observed that almost half of the women described tangible or external explanations such as poverty for depression as the perceived cause of depression, while the other half described “internalized” ones, biological causes such as physical ailments. The social causes identified by the participants were summarized in Table 3. The effect of these symptoms were also broadly categorized into two themes: the psychological effects of depression and the physiological effects of depression. The physiological effects mainly impacted the physical health of the mother and day-to-day functioning like looking after the children, tending to the domestic animals, etc. They expressed the psychological effects of depression in the form of sadness, hopelessness, anger, and lack of interest in the environment. These perceived causes were incorporated in making the case vignettes for each symptom of depression.

**Table 3:** Socio-demographic characteristics of the participants in focus group discussions (n = 28).

**Variables**	**LHW (*n* = 15)**	**PV (*n* = 13)**	**Total (*n* = 28)**
**Age**			
20–29	2	9	11
30–39	6	3	9
40–49	7	1	8
**Education**			
Matric	4	4	8
FA	6	3	9
BA/MA	5	5	11
**Work experience**			
1–5 Years	**-**	13	13
6–10 Years	4	**-**	4
>10 Years	11	**-**	11

### Step 3

In a case vignette, the aspects of the mother's life as analyzed by the IDIs were developed to seek cause and effects of symptoms. The preparation of a vignette was aided by observing the participant in a natural setting (in this study visit of the researcher to the houses of the respondents) and conducting IDI.

#### Vignette Example:

*Rashida is a 26-year-old mother of four, three girls and a boy. She lives with her husband, his parents, and siblings. Her husband is a daily wage laborer and some days he does not have any work and had a heart attack. She looks sad throughout the day. She gave birth to a baby girl her fifth child 2 years back. Most nights that she cannot sleep thinking about her ailing husband and her inability to take care of her children. Some nights, one of her children is unwell and is unable to sleep. She is kept awake by her crying. She sits up in the bed unable to lie down. She feels lonely and tired. As the night progresses she experiences worrying thoughts about her ailing husband and her frequently sick children*.

The draft of case vignettes was shared with the consultant psychologist for feedback. As the case vignettes were made for each of the nine symptoms from the IDIs, a local artist was hired to draw pencil sketches for each symptom. The pencil sketches depicted a typical Pakistani woman from a rural area wearing a kameez shalwar (local dress), the kameez, a long straight-cut, loose shirt teamed with pajamas, the loose baggy shalwar, with a rectangular scarf about 2.5 m long called the duppatta (local scarf) over her head (cultural significance of a rural woman).

### Step 4

#### Matching the Card of Symptom With the Pictorial Illustration

The majority (71.4%) of the participants in all FGDs successfully matched the card of the symptom “difficulty in sleeping” with the illustration depicting “a woman sitting in bed having difficulty in sleeping.” However, only one third (35.3%) of the participants could match cards to the illustrations showing “difficulty in household chores.” Almost half (46%) of the participants matched the symptom cards to the both pictorial illustrations depicting “suicidal ideation.” The details of the results of card matching is shown in Table 4.

**Table 4:** Analysis of card to pictorial matching (*n* = 28).

**S.No**	**Symptom**	**FGD 1 *n* = 6**	**FGD 2 *n* = 8**	**FGD 3 *n* = 7**	**FGD 4 *n* = 7**	**Total *n* = 28**
		***n*(%)**	***n*(%)**	***n*(%)**	***n*(%)**	***n*(%)**
1.	Difficulty in Sleeping	5 (83%)	6 (75%)	5 (71.4%)	4 (57.1%)	20 (71.4%)
2.	Loss of Appetite	4 (66%)	7 (87%)	4 (57.1%)	5 (71.4%)	21 (75%)
3.	Reduced Concentration	5 (83%)	5 (62.5%)	6 (85%)	2 (28%)	18 (64.2%)
4.	Helplessness	3 (50%)	2 (25%)	2 (28.4%)	2 (28%)	9 (32.1%)
5.	Suicidal Ideation	3 (50%)	5 (62%)	4 (57.1%)	3 (50%)	15 (53%)
6.	Low Energy	2 (17%)	3 (37.5%)	2 (28.4%)	2 (28%)	10 (32.1%)
7	Difficulty in Carrying Out Social Activity	2 (17%)	2 (28%)	4 (57.1%)	1 (33%)	9 (32.1%)
8.	Fatigue	4 (66%)	4 (50%)	6 (85%)	4 (57.1%)	18 64.2%)
9.	Agitation	5 (83%)	5 (62.5%)	6 (85%)	2 (28%)	18 64.2%)

#### Willingness and Acceptance to Use

Overall, the community informants were willing and able to use the tool in their daily lives. Most of the health-care workers (80%) reported that the information provided in the illustration was effective and helped them detect the mothers having the symptoms and helped them explore solutions. Although everyone was willing, not all community informants found it feasible to engage in the task fully. The Peers have a strong network in the community and were more enthusiastic to use the pictorial illustrations than LHWs. All the informants underscored the importance of providing incentives, preferably in a monetary form, upon conducting this task.

#### Facilitators and Barriers

Three broad themes were identified as facilitators to use the tool: (1) early detection of maternal depression symptoms, (2) a potential tool for psycho-education and general awareness-raising, (3) early referral. Most community informants reported illustrations as acceptable. Since LHWs serve households within a catchment area and have intimate knowledge of women's health, they indicated that using this tool will help to quickly recall and identify women in their catchment areas with similar symptoms. They also reported that they learned the usual symptoms of depression after looking at the illustrations. The key barrier to use this tool was their existing heavy workloads and the tool impinging on their time. The peers did not report this type of barrier. The key themes of the FGDs are shown in Table 5.

**Table 5:** Thematic analysis of focus group discussions (*n* = 4).

**Themes**	**Sub -Themes**	**Quotes**
Use of tool for detection	Early Detection	*I can recognize as soon as I would see the picture that there is a woman in my field who is worried and would appear like this as I have been in touch with these women for many years now (FGD02_PV02)*
	Detection despite inability of disclosure	*There are some women who feel shy and embarrassed in admitting that how can I tell that I have not eaten maybe they will think that they do not have enough to eat.If we see a picture I know that this woman is worried and is not telling me. (FGD02_PV06)*
	Early Referral	*One gets to know from the pictures that these women (similar to the pictures) are worried and need help we shall keep them in our minds and take them for a checkup. (FGD04_LHW 02)*
Use of the tool for understanding	Risk Factor for depression	*By looking at the picture one can tell that if there is a mother who has finished her errands and is sitting separately from others and does not want to talk to anyone…one gets to know that she is worried (FGD02_PV03)*
	Outcomes of depression	*This picture would indicate that there are women like this and if we detect them early so that they do not hurt themselves. (FGD01_LHW04)*
Psychosocial Awareness in Mother	Better Counseling	*If we see a woman similar to the picture we can advise her. You have eatables in front of you why are you not eating are you sick or worried? (FGD01_LHW05)*
	Better Care	*She will understand very nicely if we match it with the pictorial illustration she will understand better and look after her health better (FGD02_PV06)*
As Visual Triggers	For Health Care workers	*Yes there are in the field …there are women who work hard they have kept cattle that's why they are tired they have no time to eat food (FGD01_LHW02)*

### Step 5: Finalization of Draft

**Figure 2:**
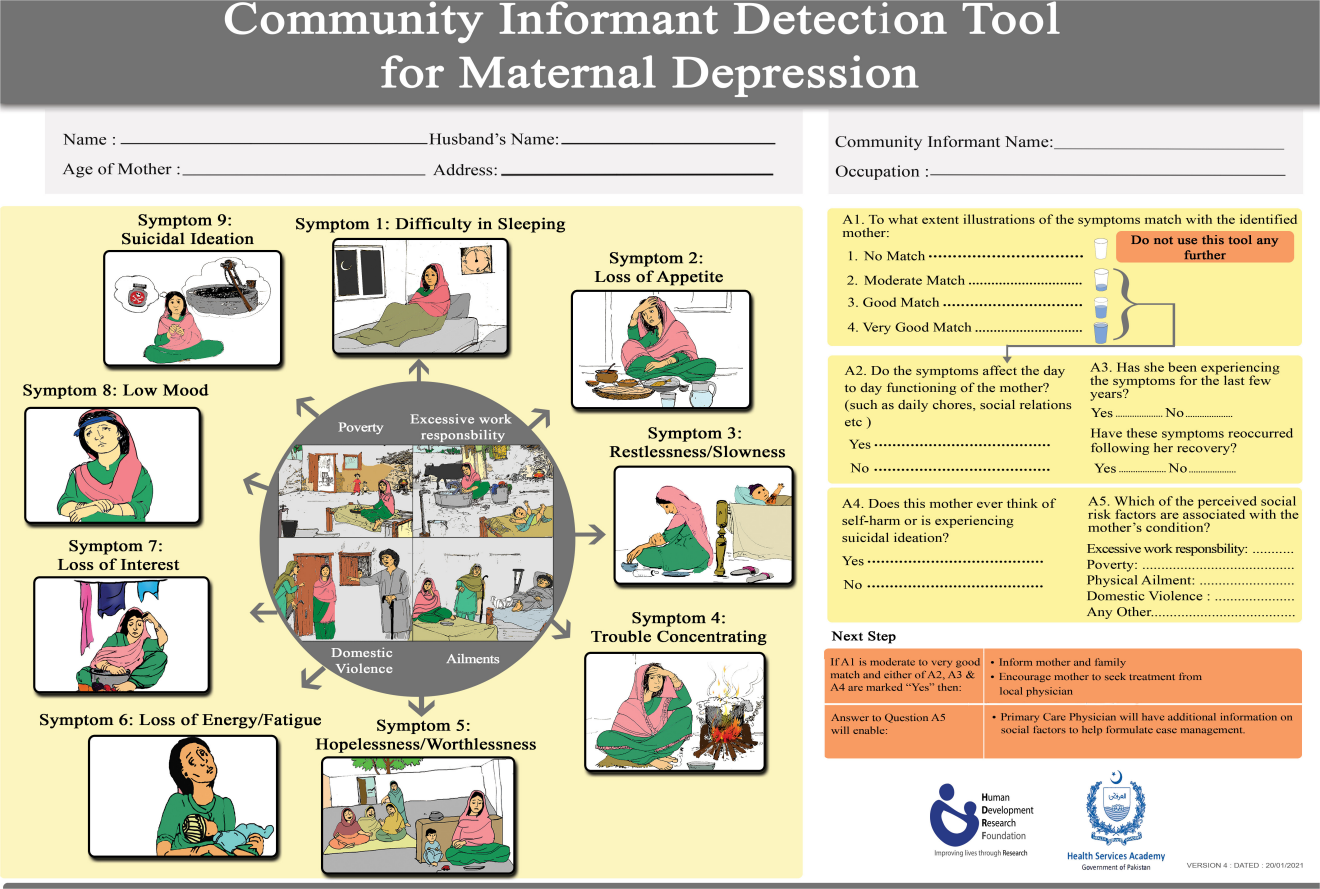
Community informant detection tool for maternal depression (CIDT-MD).

For the finalization of CIDT-MD, the pictorial illustrations were sent to a panel of mental health experts for review and feedback, to ensure that major symptoms are correctly presented in the illustrations. The experts pointed out that instead of using technical jargon in the questions relating to day-to-day functioning, chronicity, and interpersonal violence, simple statements needed to be used. They also pointed out that the analogy of glass is to be used for a better understanding of the symptom frequency. It was added as a visual analog for the informant to quickly understand and appropriately mark the extent of matching with the illustrations. They added that recurrence and chronicity of the symptom duration also needed to be mentioned. After incorporating suggestions from the expert panel, the content for the tool was finalized as shown in Figure 2. The CIDT-MD consists of a set of pictorial illustrations targeting the symptoms of depression for each likely mother. In the list the community worker will determine how much the person matches the illustration using a simple 4-point Likert scale (No match, some match, mostly match, and a complete match). The final layout of the tool had a set of colorful pictorial illustrations depicting the symptoms and yes/no response options were used for the three additional two questions about “daily functioning,” “chronicity or recurrence,” and “suicidal ideation.”

## Discussion

In the cultural adaptation of Community Informant Detection Tool for Maternal Depression (CIDT-MD), a multi-step iterative qualitative process was used. The process entailed five evidence-informed steps such as:

- The selection of an appropriate tool for adaptation through scoping review of the literature.
- A detailed formative research to explore the perceptions of key informants.
- Formulation of culturally appropriate case vignettes and easy to understand illustrations.
- Participatory feedback of end-users on the barriers and facilitators of detection.
- Finally, the approval of the detection tool by a panel of mental health experts.

The Community Informant Detection Tool for Maternal Depression (CIDT-MD) consisted of nine contextualized illustrations each depicting a symptom of depression, in addition to four illustrations encompassing the perceived cause of depression. Each likely mother in the illustration needed to be matched using a simple 4-point Likert scale (No match, some match, mostly match, and a complete match) through a glass analogy, and then three additional questions were to be answered related to daily functioning, chronicity or recurrence, and suicidal ideation, all having binary (Yes/No) responses. The rationale behind this approach proven in Nepal was that briefly trained community informants (i.e., female community health-care workers and peer volunteers) can detect mothers in need of care as they are intimately familiar with the social and cultural context of the community (17). Since this tool has the potential of being used in a collaborative care program for maternal depression, where primary care physicians will manage these cases, having additional information on issues like poverty, domestic violence, or recurrence/chronicity of depression and suicidal ideas listed by the informants will help inform the treating physician about the overall social and economic circumstances of the women. It would help the physicians to devise their depression management plan accordingly by referring to either social services or poverty alleviation programs (e.g., Benazir Income Support Program) or to family planning services on top of providing treatment for depression.

In the context of a developing country, the cultural adaptation of a detection tool is a rigorous and systematic process compared to a simple translation of a psychometric tool (18, 19). Since there is no unanimity in the best practice to be engaged for cultural adaptation, we attempted a systematic qualitative approach to satisfy the contextual needs of the target population. Such a qualitative approach has been used in the adaptation of several psychometric tools (20, 21).

After the detailed formative qualitative study to explore the idioms of depression, our key findings suggest that understanding these manifestations and cultural terms aids in the adaptation process of a detection tool considering the level of education of both the respondents and the end-users (22). Case vignettes and illustrations were created for each major symptom. A similar use of illustrations showed wider acceptability in studies conducted in Vietnam and Pakistan (23, 24). The tool contained easy to understand words used during interviews and discussions identical to an adaptation study by Arafat (25). During feedback from the end-users, it was observed that the tool was easy to use due to its simple questions, binary (Yes/No) responses, and the use of glass analogy, which were less time consuming. It was similar to the previous findings for an ideal detection tool (26). During the finalization of the draft tool with mental health experts the domains of adaptation such as comprehensibility, relevance, and motivation were all taken into consideration as the modified draft was based on these three domains (27–29).

Maternal mental health is a public health challenge and given the high prevalence of maternal depression in LMIC its early detection is a public health priority (30). When undetected and untreated, maternal depression can lead to negative health outcomes (31). Evidence indicates that a high treatment gap is a major barrier for detection in resource-constrained settings (31, 32). Similar to CIDT adaptation in Nepal we have also managed to adapt a tool, to proactively detect maternal depression. However, the accuracy of our adapted tool has been reported elsewhere (33). The evidence of accuracy of community informant led detection has already been observed in Nepal (16). Moreover, psychosocial interventions such as Thinking Healthy Program have also been demonstrated to be effectively delivered in similar low-income settings by lay health-care workers. (32).

In a resource-constrained setting as in Pakistan the LHWs can take the lead in community-based active case detection of depression as they share the same cultural and social context with the community (34). Pakistan has a well-established PHC system with rural health centers staffed by trained health workers accessible to all (35). LHWs inhabit the same rural settings (villages) they serve, being responsible for the provision of promotive and preventive health care during home visits to over a 100 households in their neighbor-hood (36). It is during these home visits that they can conduct the task of detecting maternal depression. However, research indicates that excessive workload and competing priorities for the LHWs in an overstretched PHC system may hinder early detection (37). In such cases, peer volunteers (women from the same villages sharing the same culture and life-experiences as their clientele) could be an alternative strategy proven to be effective in reducing maternal depression (32–38). Moreover, the studies conducted on maternal depression in Pakistan (39–43) all have focused on validating a screening tool, finding out the prevalence, and analyzing risk factors. To our knowledge, no study on community-based detection of maternal depression has been conducted yet and this study is the first of its kind.

The CIDT-MD is easy to administer in settings such as Pakistan where mental health systems are non-existent. Pakistan has approximately a 100 thousand LHWs covering nearly 80% of the rural population. The LHWs can become a huge resource to help active case detection for maternal depression and help link them up with health systems and eventually reduce the treatment gap of maternal depression. This culturally appropriate and easy to use detection tool is the vital first step in leveraging community-based active case detection of maternal depression. Literature indicates that screening tools require intensive training, skilled human resource, and time as they are resource-intensive in clinical and community settings (44). Using this informant based approach is an added value since it is far less resource-intensive and easy to administer through laypeople based within the community settings to help proactively detect women with symptoms of depression.

However, early detection by community informants of mothers needs to be linked to infrastructure for the continuity of mental health care through a collaborative care approach (45, 46). Evidence indicates that a collaborative care model is needed that is comprised of systematic detection, referral, and tracking the patients that can improve the health outcome, reduce depressive symptoms, and prevent the recurrence of depression (47, 48).

Our preliminary study suggests that the detection tool is acceptable, easy to comprehend, and feasible. It would be practical to integrate this detection tool into the well-established collaborative care approach rather than as a stand-alone intervention. Such integration would also be important because the same community informants are likely to detect and provide care to women. Future research is needed to assess the actual effectiveness of the community case detection procedure in accurately detecting maternal depression.

## Limitation

There are some limitations of our study. First, the adaptation of the detection tool was done only in Kallar Kahar, a rural setting that has women from a particular socio-economic background. The relevance of the adapted detection tool would be only in communities with similar socioeconomic characteristics. Despite these limitations, the findings from this study may at least be representative of similar contexts.

Second, the generalizability of this community informant detection in the absence of existing community-based workers (e.g., LHWs, Peers) might be poor because the peers and LHW work in close collaboration with the community and they are the agents of detection in the community. Similarly, the generalizability of findings to unmarried women might be limited.

## Conclusion

The Community Informant Detection Tool for Maternal Depression (CIDT-MD) is a culturally appropriate, adapted, and an acceptable tool that is easy to understand and use by the community informants in Pakistan. The next step would be to assess the accuracy of this community informant tool in detecting maternal depression by LHWs and Peers.

## Data Availability Statement

The raw data supporting the conclusions of this article will be made available by the authors, without undue reservation.

## Ethics Statement

The studies involving human participants were reviewed and approved by Institutional Ethics Review Committee, Health Services Academy, Islamabad. The patients/participants provided their written informed consent to participate in this study. Written informed consent was obtained from the individual(s) for the publication of any potentially identifiable images or data included in this article.

## Author Contributions

NA, SS, SM, and SK conceptualized the study. SM wrote the first draft. SS, NA, and SK critically reviewed the article and gave their input. NA and SS trained and supervised the data collectors AT, MT, and SM. WR and SM analyzed the qualitative data under the supervision of NA. All authors read and approved the final article.

## Conflict of Interest

The authors declare that the research was conducted in the absence of any commercial or financial relationships that could be construed as a potential conflict of interest.
